# LegC3, an Effector Protein from *Legionella pneumophila*, Inhibits Homotypic Yeast Vacuole Fusion *In Vivo* and *In Vitro*

**DOI:** 10.1371/journal.pone.0056798

**Published:** 2013-02-20

**Authors:** Terry L. Bennett, Shannon M. Kraft, Barbara J. Reaves, Joji Mima, Kevin M. O’Brien, Vincent J. Starai

**Affiliations:** 1 Department of Microbiology University of Georgia, Athens, Georgia, United States of America; 2 Infectious Diseases University of Georgia, Athens, Georgia, United States of America; 3 Institute for Protein Research, Osaka University, Osaka, Japan; University of Minnesota, United States of America

## Abstract

During infection, the intracellular pathogenic bacterium *Legionella pneumophila* causes an extensive remodeling of host membrane trafficking pathways, both in the construction of a replication-competent vacuole comprised of ER-derived vesicles and plasma membrane components, and in the inhibition of normal phagosome:endosome/lysosome fusion pathways. Here, we identify the LegC3 secreted effector protein from *L. pneumophila* as able to inhibit a SNARE- and Rab GTPase-dependent membrane fusion pathway *in vitro*, the homotypic fusion of yeast vacuoles (lysosomes). This vacuole fusion inhibition appeared to be specific, as similar secreted coiled-coiled domain containing proteins from *L. pneumophila*, LegC7/YlfA and LegC2/YlfB, did not inhibit vacuole fusion. The LegC3-mediated fusion inhibition was reversible by a yeast cytosolic extract, as well as by a purified soluble SNARE, Vam7p. LegC3 blocked the formation of trans-SNARE complexes during vacuole fusion, although we did not detect a direct interaction of LegC3 with the vacuolar SNARE protein complexes required for fusion. Additionally, LegC3 was incapable of inhibiting a defined synthetic model of vacuolar SNARE-driven membrane fusion, further suggesting that LegC3 does not directly inhibit the activity of vacuolar SNAREs, HOPS complex, or Sec17p/18p during membrane fusion. LegC3 is likely utilized by *Legionella* to modulate eukaryotic membrane fusion events during pathogenesis.

## Introduction

Protein and membrane trafficking events during eukaryotic growth are regulated at many steps, such as the selection of the appropriate cargo, the proper construction, budding, and targeting of secretory vesicles to the appropriate membrane-bound compartments, and the final fusion of those transport vesicles with their target membranes. As an essential step in all trafficking events including neurotransmission, exocytosis, and hormone secretion, intracellular membrane fusion events are catalyzed by a highly conserved set of core machinery consisting of soluble NSF attachment protein receptors (SNAREs) [1], [2], Rab-family GTPases [3], [4], Sec1-Munc18 (SM) family member proteins [5], [6], [7], [8], membrane tethering factors [9], [10], and the SNARE protein chaperones, *N*-ethylmaleimide-sensitive factor (NSF) and soluble NSF attachment protein (α-SNAP) [11], [12], [13], [14]. The biochemical characterization of these proteins and the mechanisms by which this machinery regulates and drives the mixing of bilayer lipid membranes has been quite extensive, leading to a general fusion model in which SNARE proteins, engaged in pre-fusion cis-SNARE complexes, are disassembled and freed via ATP hydrolysis and NSF/α-SNAP activity [15], [16]. Membrane-associated, GTP-bound Rab-family GTPases recruit essential effectors such as membrane tethering complexes [10] and lipid modifying enzymes, such as phosphatidylinositol 3-kinase [17], thus enabling the initial contact between fusing compartments. Free cognate SNARE proteins assemble into tight, parallel, 4-helical bundles across these membranes (trans-SNARE complexes, or SNAREpins) [18], [19], [20], [21], fusion proteins and fusion-competent lipids organize into microdomains at the site of fusion [22], [23], [24], and lipid bilayer and non-leaky lumenal content mixing proceeds.

The intracellular bacterial pathogen *Legionella pneumophila* causes a severe form of pneumonia known as Legionnaires’ disease. As a prerequisite for intracellular survival, *L. pneumophila* requires a functional Dot/Icm Type IV secretion system to inject more than 250 protein effectors into the host cell [25], [26], [27], [28], [29], [30]. These effector proteins quickly hijack and manipulate normal host cell processes, allowing the bacterium to evade lysosomal degradation through the manipulation of normal phagocytic trafficking pathways [31], [32]. Upon phagocytic uptake into alveolar macrophages [33], or a diverse group of protist hosts like the amoeba *Hartmanella vermiformis* [34], *L. pneumophila* prevents fusion of the bacteria-laden phagosome (also called the *Legionella*-containing vacuole, or LCV) with early endosomal compartments, as shown by the inability of the phagosome to accumulate the early endosomal small GTPase Rab5 [35]. *Legionella* directly blocks fusion of the LCV with lysosomal compartments through the use of both the Dot/Icm Type IV secretion system, and possibly another secretion-independent pathway dependent upon outer membrane shedding [36]. Membrane-bound compartments containing live *Legionella* also exclude the late endosomal/lysosomal markers LAMP-1 and Rab7 [37], thereby confirming the inhibition of normal phagosome:lysosome delivery pathways. In parallel with inhibiting phagolysosomal fusion steps, *L. pneumophila* modulates other normal membrane trafficking events, as seen by the extensive modification of the LCV with vesicles derived from host endoplasmic reticulum, as shown by electron microscopy [32]. In order to regulate the ER membrane-dependent “decoration” of the LCV, *L. pneumophila* recruits the ER SNARE Sec22b on ER-derived vesicles into aberrant SNARE complexes with plasma membrane syntaxins present on the LCV [38]. Furthermore, *L. pneumophila* modulates the GTPase activity of a host Rab-family GTPase critical for ER-Golgi transport, Rab1, through the activity of a secreted multifunctional effector protein, DrrA/SidM, which associates with the phagosomal membrane through its intrinsic PI(4)P-binding activity. DrrA/SidM then recruits and AMPylates Rab1, thereby diminishing the Rab’s affinity for normal host Rab GTPase activating proteins, and displays guanine nucleotide exchange activity on Rab1 (reviewed in [39]). To further modulate Rab1 activity, *Legionella* produces a secreted Rab1 GTPase-activating protein (GAP), LepB, allowing *Legionella* to now fully control a Rab GTPase-dependent trafficking pathway within the host [40].

Due to the fact that *Legionella* can invade and survive within a wide range of phylogenetically-diverse amoeba and human alveolar macrophages, it is likely that the protein and organellar targets of the secreted *Legionella* effector proteins are evolutionarily conserved among eukaryotes. In support of this hypothesis, the budding yeast *Saccharomyces cerevisiae* has long been used as a model for general eukaryotic processes, and has been successfully used to identify and characterize secreted effector proteins from pathogenic intracellular prokaryotes such as *L. pneumophila*, *Salmonella enterica*, and *Chlamydia trachomatis* (reviewed in [41]). Recently, three proteins from *L. pneumophila –* LegC3, LegC7/YlfA, and LegC2/YlfB – have been shown to cause visible endomembrane system defects and vacuolar protein mis-sorting upon expression in *S. cerevisiae*, suggesting that these proteins play a role in modulating eukaryotic protein trafficking and membrane fusion dynamics [42]. Of these three proteins, only LegC3 was found to highly fragment the vacuolar membrane. Yeast vacuoles (similar to mammalian lysosomes) use a core set of membrane fusion machinery to maintain the appropriate copy number of the organelle, including SNAREs (Vam3p, Vti1p, Vam7p, and Nyv1p), a Rab-family GTPase (Ypt7p), α-SNAP/NSF (Sec17/18p), and a multisubunit tethering complex (HOPS complex) (reviewed in [43]). Inhibition of this fusion machinery by mutation *in vivo* leads to vacuolar fragmentation [44], similar to the defect previously seen in *S. cerevisiae* strains expressing the *L. pneumophila* LegC3 protein [42]. Therefore, we examined LegC3’s ability to directly inhibit the fusion of yeast vacuoles *in vivo* and *in vitro*. In this work, we show that LegC3 can inhibit yeast vacuole fusion *in vitro*, likely by disrupting the formation of a critical trans-SNARE complex intermediate, even though LegC3 does not associate directly with the fusion-competent vacuolar SNARE complex.

## Materials and Methods

### Yeast Strains, Plasmid Constructions, and Genetic Manipulations

Yeast strains BJ3505 (*MAT*a *ura3*-*52 trp1*-Δ*101 his3*-Δ*200 lys2*-*801 gal2 (gal3) can1 prb1*-Δ*1.6R pep4*::*HIS3*) [45] and DKY6281 (*MAT*a *ura3*-*52 leu2*-*3*,*112 trp1*-Δ*901 his3*-Δ*200 lys2*-*801 suc2*-Δ*9 pho8*::*TRP1*) [46] were used for vacuole production. Yeast strain BY4742 (*MAT*α *his3*Δ*1 leu2*Δ*0 lys2*Δ*0 ura3*Δ*0*) was used for all *in vivo* growth studies.

To construct galactose-inducible yeast expression vectors for *Legionella* effectors, the *LEGC3* gene was PCR amplified from the *Legionella pneumophila* strain Lp02 (Philadelphia-1 *hsdR rpsL thyA*) chromosome (a generous gift from Dr. Michele Swanson, University of Michigan), using the primer pair LegC3-KpnI and LegC3-XbaI (Table 1). *LEGC2* was amplified with the primer pair LegC2-KpnI and LegC2-XbaI; *LEGC7* was amplified with the primer pair LegC7-KpnI and LegC7-XbaI. These amplicons were digested with KpnI and XbaI and ligated separately into plasmid pYES2/NT C (Invitrogen), digested with the same enzymes. The resultant plasmids were named pVJS40, pVJS51, and pVJS52, respectively. The open reading frames were cloned in-frame with the vector-encoded N-terminal hexahistidine tag.

**Table 1:** Primers used in this study.

Primer Name	Sequence*
LegC3-KpnI	5′-GTAGAA*GGTACC*CGTGATTATGTTTTTGGCCAAC-3′
LegC3-XbaI	5′-GGTGGT*TCTAGA*GCTCCATTGAAATTTTATTGACAG-3′
LegC2-KpnI	5′-GTAGAA*GGTACC*CATGACAGACACTCCAAAAGC-3′
LegC2-XbaI	5′-GGTGGT*TCTAGA*TGGTTGACCGCACGTGATAAG-3′
LegC7-KpnI	5′-GTAGAA*GGTACC*CATGGCTACTAATGAAACAGAGC-3′
LegC7-XbaI	5′-GGTGGT*TCTAGA*GCCTGATTCTTCTTCCTTAAATC-3′
LegC3-NdeI	5′-GGTGGT*CATATG*ATTATGTTTTTGGCCAAC-3′
LegC3ΔTM-SapI	5′-GGTGGT*TGCTCT*TCCGCAGCCCAAGCGATGACGTAGG-3′
LegC2ΔTM-BsmI	5′- GGTGGTGG*GAATGCT*GAAAATCATGTTCAAAAAGAAG-3′
LegC2-EcoRI	5′- GGTGGT*GAATTC*TAACCTGTGGAGTTTGAG-3′
LegC7ΔTM-BsmI	5′-TAATAAGG*GAATGCT*GACGACCACCATACATGCAATG-3′
LegC7-EcoRI	5′-GGTGGT*GAATTC*TTAATTGACTAAAGCAATAGTTTGTCTATC-3′
Gyp1-forward	5′-TGAACTCCATCATCCAGC-3′
Gyp1-46 reverse	5′-CAGCCAGTGCGACGTAGC-3′

* Italics denote introduced restriction sequences.

To create vectors for the recombinant expression *Legionella* effector proteins, the *LEGC3* gene was amplified from the *L. pneumophila* Lp02 chromosome using the primer pair LegC3-NdeI and LegC3ΔTM-SapI, creating an amplicon encoding for residues 1-370 of LegC3. This fragment was digested with NdeI and SapI, and cloned into the chitin-affinity purification system vector, pTYB1 (New England Biolabs), resulting in plasmid pVJS39. The *LEGC2* gene was amplified from the *L. pneumophila* Lp02 genome using the primer pair LegC2ΔTM-BsmI and LegC2-EcoRI, encoding residues 123-405. *LEGC7* was amplified with the primer pair LegC7ΔTM-BsmI and LegC7-EcoRI, encoding for residues 133-425 of LegC7. These *LEGC2*ΔTM and *LEGC7*ΔTM amplicons were digested with BsmI and EcoRI, and ligated into pTYB12 (New England Biolabs) digested with the same enzymes, creating plasmids pVJS46 and pVJS47, respectively.

### Vacuole Isolation and in vitro Fusion Assay

Vacuoles were prepared from BJ3505 (*pep4*Δ) and DKY6281 (*pho8*Δ) yeast strains on discontinuous Ficoll gradients, as described [47]. Standard vacuole fusion assays (30 µl final volume, 27°C, 90 minutes) contained 3 µg each BJ3505 and DKY6281 vacuoles (6 µg total), 20 mM piperizine-*N,N’*-bis(2-ethanesulfonic acid) (PIPES)-KOH (pH 6.8), 200 mM sorbitol, 10 µM coenzyme A, 125 mM KCl, 5 mM MgCl_2_, 815 nM Pbi2p (I_2_^B^), 1 mM ATP, 1 mg/ml creatine kinase, and 29 mM creatine phosphate. Where indicated, low-salt fusion reactions for LegC3ΔTM, LegC2ΔTM, and LegC7ΔTM studies contained 75 mM KCl, instead of 125 mM. After 90 minutes, fusion reactions were assayed for active Pho8p alkaline phosphatase as a measure of vacuole fusion, except that CaCl_2_ was omitted from the standard development solution. Units of fusion are reported as nmol *p*-nitrophenylate formed min^−1^ µg *pep4*Δ vacuole^−1^.

### Recombinant LegC3ΔTM, LegC2ΔTM, and LegC7ΔTM Purifications

For the purification of LegC3ΔTM protein, pVJS39 was transformed into *Escherichia coli* strain Rosetta™2(DE3) (Novagen). The resultant strain was grown 16 h at 37°C in 25 ml LB supplemented with 100 µg/ml ampicillin. This culture was used to inoculate 1 L Terrific Broth supplemented with 100 µg/ml ampicillin, and grown at 30°C until OD_600_ = 0.6-0.8. Isopropyl β-D-1-thiogalactopyranoside (IPTG) was added to 1.0 mM, and outgrowth continued for 5 hr at 30°C. Cells were harvested by centrifugation (JLA-10.500 rotor, 5000 rpm, 10 min) and completely suspended in chitin binding buffer (20 mM Hepes-KOH, pH 8.0, 0.5 M NaCl, 0.1% (v/v) Triton X-100, 1 mM EDTA). LegC3ΔTM protein was purified via chitin-affinity chromatography via the manufacturer’s instructions, with modifications: after protein binding and initial 2-column volume (CV) wash with chitin binding buffer, a 15-CV wash was performed (2×CV, 2×CV, 11×CV) with chitin binding buffer lacking Triton X-100. Eluted LegC3ΔTM protein was dialyzed into PS buffer (20 mM PIPES-KOH, pH 6.8, 200 mM sorbitol) containing 300 mM KCl.

LegC2ΔTM and LegC7ΔTM were purified as LegC3ΔTM, but with minor modifications. IPTG induction was performed at OD_600_ = 1.5, and post-induction growth continued 16 h at 18°C. During the final elution/intein-cleavage from the chitin beads, the resin was placed at 16°C for 16 h. All other wash steps and buffers were as for LegC3ΔTM.

### Reagent Preparation

Purified recombinant yeast proteins Pbi2p [48], Sec17p [49], Sec18p [15], and Gdi1p [50] were isolated as previously described. Inhibitory antibodies against Vam3p and Sec17p were purified from serum, as previously described [15], [51], and routinely used in fusion assays at 450 nM and 190 nM, respectively.

Recombinant Vam7p SNARE protein was purified as previously described, except that only intein cleavage was carried out for purification [49]. The cytosolic domains of the Vti1p (residues 1-194), Vam3p (residues 1-259), and Nyv1p (residues 2-231) were purified as previously described [52].

The catalytically active domain of the Gyp1 protein (Gyp1-46, [53]) was amplified from the BY4742 chromosome with the primer pair Gyp-1 forward and Gyp1-46 reverse. This amplicon was ligated into pTYB2 (New England Biolabs), which had been digested with NdeI and SmaI. Prior to ligation, the resultant overhang in the plasmid NdeI site was filled-in with DNA polymerase I (Klenow fragment), thereby generating pVJS23. This plasmid was transformed into *E. coli* strain Rosetta™2(DE3) (Novagen), and produced a Gyp1-46p-CBD fusion protein that was purified via chitin affinity chromatography and intein cleavage. Purification followed the manufacturer’s directions, except that Triton X-100 was left out of the wash and elution buffers. Intein-cleaved Gyp1-46 was dialyzed into PS buffer containing 125 mM KCl.

Purified yeast HOPS complex [54] and antisera against Vti1p, Vam3p, Vam7p, Nyv1p, Vps33p, Sec17p, and Sec18p were generous gifts from Dr. William Wickner (Dartmouth Medical School). Antibodies against purified LegC3ΔTM protein were raised in rabbits via standard protocols (Rockland Immunochemicals, Inc.).

### GFP Release (Lysis) Assay

Lysis of vacuoles was assayed via measuring the release of a lumenal GFP protein after fusion, as previously described [55]. Briefly, fluorescent vacuoles were isolated from yeast strain VSY39, a protease-deficient BJ3505 background constitutively expressing GFP targeted to the vacuolar lumen via fusion to an N-terminal signal sequence from the *Saccharomyces carlsbergensis* α-galactosidase, *MEL1* [56]. These vacuoles were premixed in a 1∶1 (fluorescent *pep4*Δ/nonfluorescent *pho8*Δ) ratio before the addition of other reaction components. Standard vacuole fusion reactions were used for the GFP release assay, with the following modifications: reactions contained 0.1×protease inhibitor mixture (50×stock: 13 µg/ml leupeptin, 25 µg/ml pepstatin A, and 5 mM Pefabloc SC) to stabilize GFP after vacuolar release. Each reaction was performed on a 3×scale (90 µl) under low-salt conditions, and fused for 60 min at 27°C. All reactions not containing LegC3ΔTM protein contained the appropriate buffer control. Fusion detection and vacuolar pellet/supernatant separation was performed as described [55], [57]; GFP signal in each 20 µl vacuole membrane pellet or supernatant sample was measured in a BioTek SynergyMX plate reader (BioTek, Winooski, VT) (λ_ex_ = 462 nm; λ_em_ = 510 nm, Read Height = 8.00 mm, gain = 100). The amount of GFP released (lysis) was calculated as: RFU supernatant *1.2/(RFU supernatant+RFU pellet) * 100%.

### Preparation of SNARE Proteoliposomes

Full-length, untagged vacuolar SNAREs, which include Vam3p, Vam7p, Vti1p, and Nyv1p, were purified as described [58], except that 100 mM β-octylglucoside (β-OG) (Nacalai, Kyoto, Japan), instead of 40 mM CHAPS (Roche), was used as detergent for purifying the SNAREs. Proteoliposomes bearing purified vacuolar SNAREs were prepared as described [58] with modifications. Vacuole-mimic lipid mixes contain POPC [43 or 45% (mol/mol) for donor or acceptor liposomes, respectively], POPE (18%), soyPI (18%), POPS (4.4%), POPA (2.0%), CL (1.6%), ERG (8.0%), DAG (1.0%), PI(3)P (1.0%), and fluorescent lipids (1.5% each of NBD-PE and Rh-PE or 1.0% dansyl-PE for donor or acceptor proteoliposomes, respectively). The detergent-lipid-SNARE mixed micellar solutions in RB500 (20 mM HEPES-NaOH, pH 7.4, 500 mM NaCl, 10% glycerol) with 100 mM β-OG were extensively dialyzed against RB500 to remove β-OG, followed by centrifugation [TLS-55 (Beckman), 166,000×*g*, 2 h, 4°C] in discontinuous Histodenz density gradients to further purify SNARE proteoliposomes.

### SNARE-dependent Lipid Mixing Assay

Liposome mixing was carried out as in [58], with some modifications. Briefly, standard reaction mixtures in RB150 (30 µl final) were mixed on ice, containing donor liposomes (33.3 µM lipids), acceptor liposomes (266.7 µM lipids), 1 mM MgCl_2_, 0.5 mM ATP, Sec17p (0.3 µM), and Sec18p (0.25 µM, monomer). Purified α-Vam3p IgG (1.0 µM), HOPS complex (35 nM) and LegC3ΔTM (3.5µM) were added, where indicated. Reactions lacking LegC3ΔTM or HOPS complex contained equivalent volumes of the appropriate no-protein buffers.

Reaction mixtures lacking Sec17p, Sec18p, and HOPS were added to a black 384-well low volume, round bottom plate (Corning), and incubated in a SynergyMX multimode plate reader (BioTek) equilibrated to 27°C. Detection sensitivity was set to a value of 70, and the top probe vertical offset was set to 10.00 mm. During an initial 10 min incubation, fluorescence emission was measured (λ_ex = _462 nm, λ_em_ = 538 nm) every 30 s to establish a fluorescence baseline. After 10 min, the indicated protein mixtures were added to the lipids, gently mixed, and fluorescence measurements continued every 30 s over 60 min.

### Statistical Analysis

Statistical analysis was performed within the Prism® software package (GraphPad Software, v. 5.0c). Column statistics were performed via a 1-way ANOVA Repeated Measures test and Tukey’s Multiple Comparison post-test. Where noted in figures, ns = P>0.05 (not significant); (*) = P ≤ 0.05; (**) = P ≤ 0.01; (***) = P ≤ 0.001.

## Results

### LegC3 Expression in Yeast Disrupts Vacuole Morphology and Function

**Figure 1:**
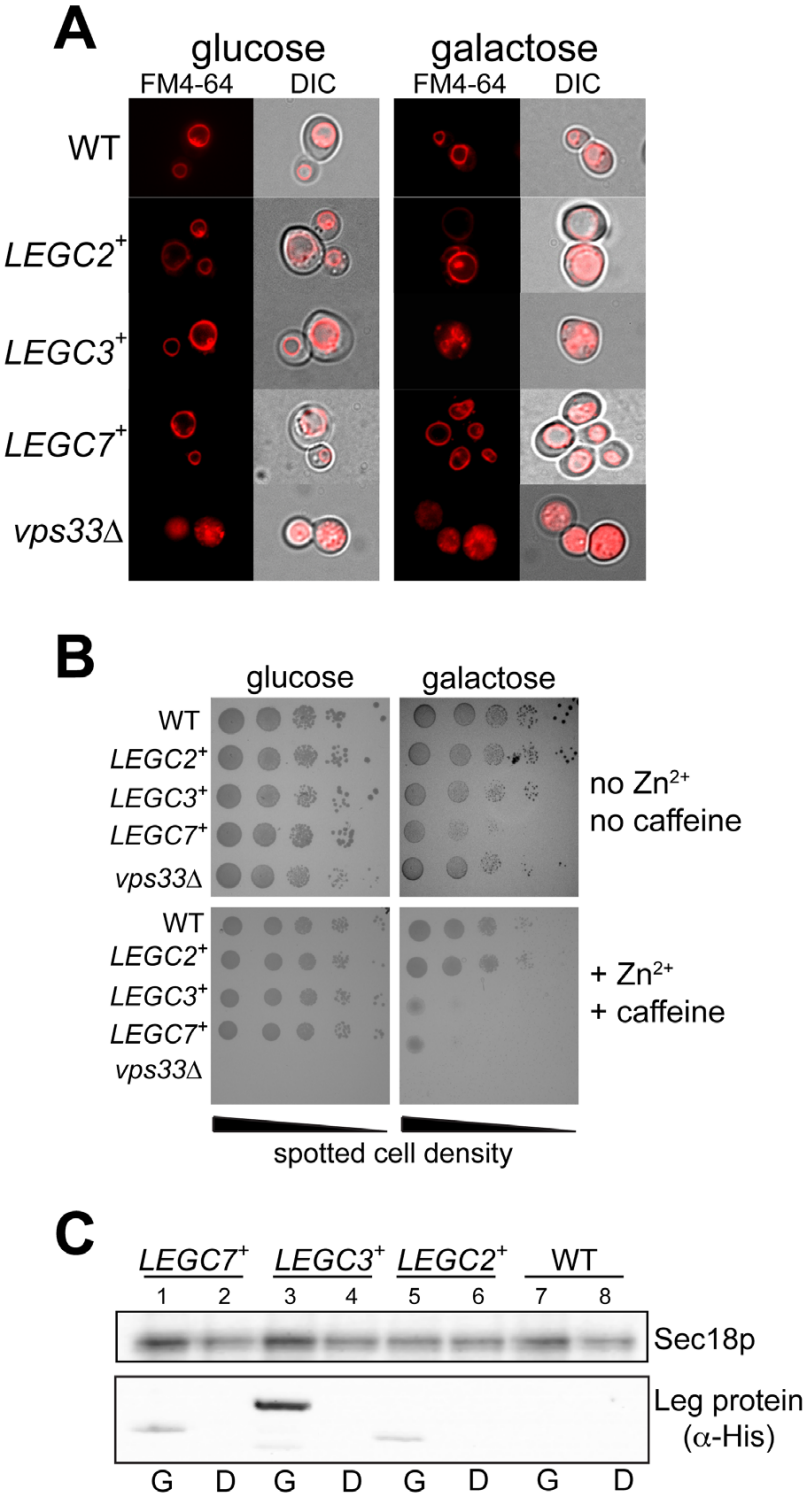
Expression of LegC3 in *S. cerevisiae* disrupts normal vacuole function *in vivo*. (***A***) BY4742 yeast strains harboring either the galactose-inducible control vector pYES2/NT C, pVJS51 (*LEGC2* ^+^), pVJS40 (*LEGC3^+^*), pVJS52 (*LEGC7^+^*), or deletions in *VPS33* (*vps33*Δ) were subcultured from saturated CSM-uracil medium supplemented with 2% glucose, to equal cell densities (0.25 OD_600_ units/5 ml) in either fresh CSM-uracil/2% glucose (left panels) or CSM-uracil/2% galactose (right panels) medium. Cells were grown for 20 hours at 30°C with shaking, loaded with the fluorescent vacuolar membrane stain FM4-64 (Invitrogen), and vacuole morphology was visualized via epifluorescent microscopy [91]. (***B***) These same 5 strains were spotted (10 µl) in 10-fold serial dilutions, made in sterile 0.9% NaCl from a starting culture of OD_600_ = 1.0. Plates contained CSM-uracil and either 2% glucose (left panels) or 2% galactose (right panels), and were supplemented with either 7.5 mM caffeine/5 mM ZnCl_2_ (bottom panels), or not supplemented (top panels). Glucose plates were incubated for 72 h at 30°C; galactose for 96 h. *(* ***C*** *)* 3.0 OD_600_ units of strains from *(* ***B*** *)* were harvested by centrifugation, and total protein was extracted from these cells as in [92]. 20 µl of each extract was separated by SDS-PAGE, and immunoblotted for the presence of Sec18p (loading control) or Leg proteins (via α-His mAb). Glucose growth conditions are labeled (D), galactose growth conditions are labeled (G).

A previous study has identified membrane trafficking and *v*acuolar *p*rotein *s*orting (*vps*) defects caused by the ectopic expression of the *Legionella pneumophila* LegC3 protein in *Saccharomyces cerevisiae* [42]. To further study the effects of LegC3 on intracellular membranes in *S. cerevisiae,* we constructed a yeast expression plasmid that allows for the galactose-inducible expression of LegC3. Yeast strains harboring this plasmid show distinct fragmentation of the yeast vacuolar membrane when grown on media containing 2% galactose, but not under glucose growth conditions (Figure 1A). This vacuole morphology difference is in striking contrast to vector controls (top panels), and is in agreement with this previous report describing the effects of LegC3 in yeast. Additionally, this vacuolar fragmentation is similar to that seen in yeast strains lacking the Vps33p subunit of the two normal endosomal/vacuolar membrane tethering complexes, CORVET and HOPS, which is required for normal vacuolar morphology, vacuolar protein sorting, and homotypic vacuole fusion *in vivo* and *in vitro* [5], [59]. In contrast, and in confirmation of a previous study [42], yeast strains harboring vectors expressing the similar coiled-coiled domain transmembrane effector proteins from *Legionella*, LegC2/YlfB and LegC7/YlfA, did not show strikingly abnormal vacuolar morphologies under these same conditions, suggesting that this vacuolar fragmentation activity of LegC3 at the vacuole is likely specific.

While the vacuolar compartment is not essential for yeast survival, it is known that normal vacuole function in yeast is required to support growth in the presence of external stressors, such as high concentrations of zinc chloride and caffeine [60], [61]. We therefore assayed whether yeast strains expressing LegC3 protein could survive on plates containing 7.5 mM ZnCl_2_ and 5 mM caffeine, levels that wild-type yeast strains can typically tolerate (Figure 1B, WT). Correspondingly, *vps33*Δ strains do not grow in the presence of ZnCl_2_ and caffeine (Fig. 1B), highlighting the requirement for normal vacuole function and *in vivo* homotypic vacuolar fusion for growth on this medium. Similar to *vps33*Δ strains, galactose-mediated expression of LegC3 causes a growth defect in the presence of zinc and caffeine, indicative of the inhibition of normal vacuole function (Fig. 1B, compare upper and lower right panels) and possibly of the inhibition of homotypic vacuole fusion *in vivo*. Under these same conditions, yeast strains expressing LegC2/YlfB or LegC7/YlfA do not show enhanced sensitivity to zinc and caffeine, although LegC7/YlfA causes a severe growth defect on galactose-only media, in support of its initial identification as a yeast lethal factor [27]. The absence of vacuolar fragmentation and growth phenotypes of LegC2- and LegC7-expressing strains are not a direct result of a lack of protein expression (Figure 1C).

### LegC3 Directly Inhibits Homotypic Vacuole Fusion in vitro

Because severe vacuolar fragmentation in *Saccharomyces* can be a hallmark of homotypic vacuole fusion defects due to the unbalanced continuation of vacuolar fission, and since yeast strains expressing LegC3 displayed growth defects on zinc and caffeine, similar to strains known to be defective in homotypic vacuole fusion, we postulated that LegC3 might be directly blocking homotypic vacuole fusion. To assay this possibility in a more purified system, we employed a cell-free model system that allows us to directly measure the SNARE-dependent, Rab GTPase-dependent homotypic fusion of yeast vacuoles *in vitro*.

We routinely assay the fusion between vacuoles isolated from two different yeast strains. One strain produces a vacuole that contains the major, vacuole membrane-bound, phosphatase, Pho8p, yet lacks the lumenal proteases required for the proper maturation of Pho8p into its catalytically-active form. The other yeast strain produces a vacuole that contains the full complement of vacuolar proteases, yet lacks the Pho8p protein. While neither of these vacuoles displays active Pho8p-dependent phosphatase activity, lipid mixing and lumenal content exchange during membrane fusion allows for the activation of Pho8p, and this activity can be measured colorimetrically [47]. This system has been successfully employed to discover and characterize many of the critical conserved regulators of eukaryotic intracellular membrane fusion, including SNARE proteins, membrane tethering factors, SNARE chaperones, and Rab-family GTPases (review in [43]).

**Figure 2:**
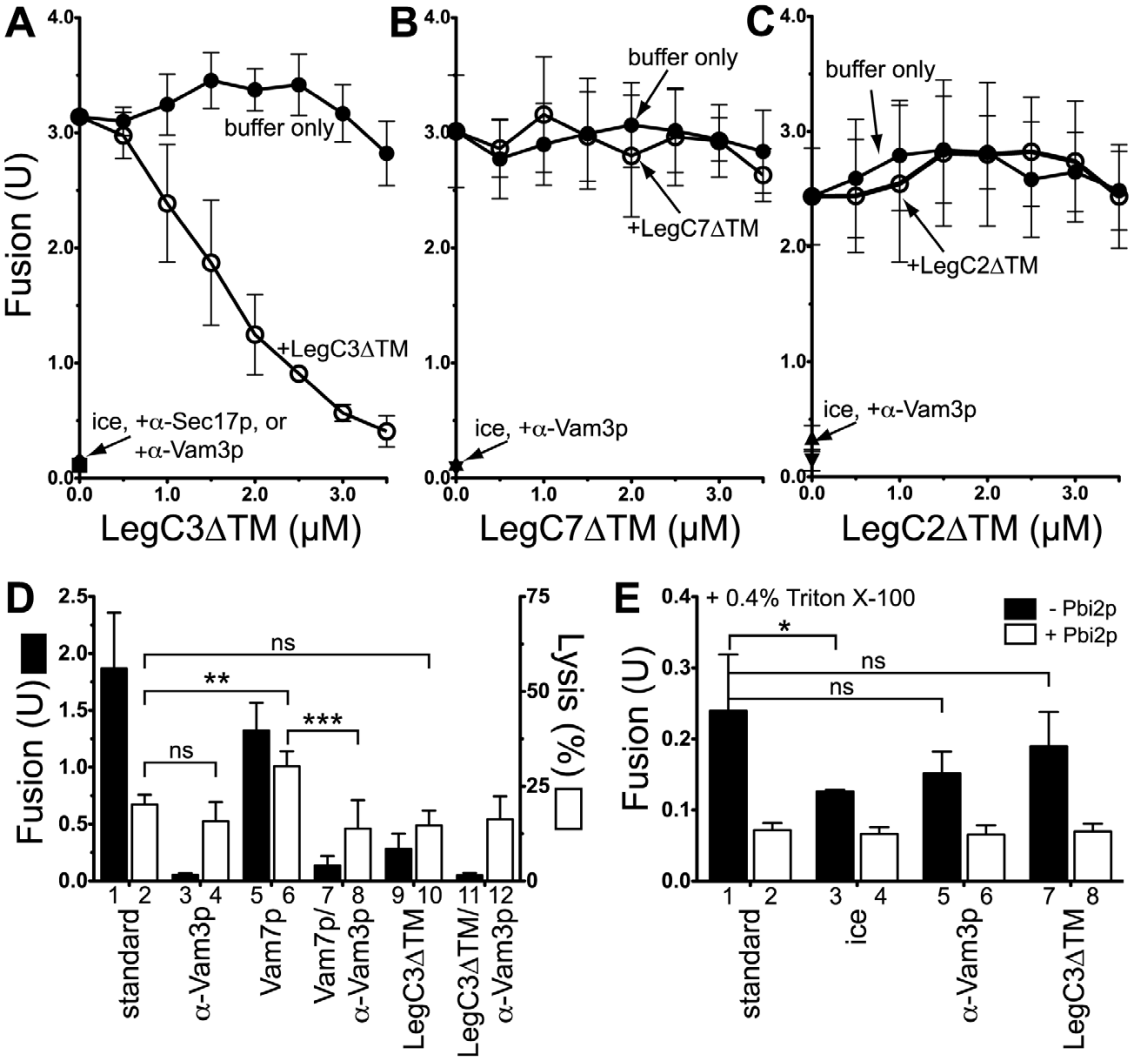
LegC3ΔTM, but not LegC2ΔTM or LegC7ΔTM, inhibits vacuole fusion *in vitro*. Low-salt, 90 min vacuole fusion assays were performed with vacuoles harvested from BJ3505 and DKY6281 (Materials and Methods), and in the presence of increasing concentrations of purified LegC3ΔTM (***A***), LegC7ΔTM (***B***), or LegC2ΔTM (***C***) proteins (open circles). To control for increased KCl additions, appropriate volumes of no-protein buffer (20 mM Pipes-KOH pH 6.8, 200 mM sorbitol, 300 mM KCl) was added to each “buffer only” reaction (closed circles). Results are standard deviation from mean, *n* = 3. *(* ***D*** *)* GFP release (lysis) assays were performed as described in Materials and Methods, and contained 1 µM recombinant Vam7p or 3.5 µM LegC3ΔTM, where noted. Fusion (black bars) and GFP release (white bars) were measured for each reaction condition. *(* ***E*** *)* Standard, low-salt vacuole fusion reactions were performed in the presence of 0.4% Triton X-100, either in the absence (black bars) or presence (white bars) of the soluble proteinase B inhibitor, Pbi2p. LegC3ΔTM was used at 3.5 µM, and reactions lacking LegC3ΔTM contained the same volume of protein-free buffer. Error for all panels is standard deviation from mean, *n* = 3.

The predicted structure of LegC3 shows that the C-terminus contains two putative transmembrane domains, and possibly three coiled-coil regions in the N-terminus [42]. Further bioinformatics has suggested that the coiled-coil regions in the N-terminus may be “SNARE-like,” even in the absence of sequence homology, and therefore could be directly involved in modulating SNARE protein assembly or function [62]. To test the possibility that LegC3 could directly interfere with yeast homotypic vacuole fusion, we purified the *L. pneumophila* LegC3 protein lacking its predicted C-terminal transmembrane domains (LegC3ΔTM), and added increasing concentrations to the *in vitro* vacuole fusion assay. As seen in Figure 2A, LegC3ΔTM inhibits the homotypic fusion of vacuoles in a dose-dependent manner (open circles), with 3.5 µM LegC3ΔTM approaching the inhibitory potency of the well-established vacuole fusion inhibitors, α-Vam3p and α-Sec17p. While LegC3 protein required its transmembrane domains for proper trafficking to – and fragmentation of – the yeast vacuole *in vivo* ([42], and data not shown), these transmembrane domains are not absolutely required for membrane fusion inhibition.

To test whether this *in vitro* membrane fusion-inhibiting activity of LegC3 was a general property of secreted coiled-coiled domain-containing proteins from *L. pneumophila*, we chose to assay the activities of the two additional predicted coiled-coiled proteins of *L. pneumophila*, LegC7/YlfA and LegC2/YlfB. Both LegC7/YlfA and LegC2/YlfB are predicted to have extensive coiled-coil regions in their C-termini, and both contain a putative single transmembrane domain within their N-terminus. While neither of these proteins was shown to cause vacuolar fragmentation like LegC3 *in vivo* (Figure 1 and [42]), the presence of LegC7 in *S. cerevisiae* was shown to cause the extensive secretion of a protein trafficked to the vacuole lumen (CPY-invertase). This phenotype highlights LegC7’s potential role in perturbing eukaryotic membrane trafficking and dynamics pathways, specifically those pathways linked to normal vacuolar traffic. In addition to LegC3, LegC7 and LegC2 have a similar overall domain structure to the *Chlamydia trachomitis* IncA protein; this SNARE-like molecule is proposed to be involved in directly modulating SNARE complex assembly and homotypic fusion events of the chlamydial inclusion [62], [63], [64]. However, neither the purified LegC2ΔTM nor LegC7ΔTM proteins were able to inhibit the *in vitro* vacuole fusion assay (Fig. 2 B-C, comparing closed and open circles), over the same molar concentrations used for LegC3ΔTM protein. These results suggest that the vacuole fusion inhibitory activity of the LegC3ΔTM protein may show some specificity for a vacuolar receptor, and this activity is not a result of simply being a SNARE-like, coiled-coiled protein from *L. pneumophila*.

To ensure that the inhibitory activity of LegC3ΔTM was not due to the disruption of vacuolar integrity prior to fusion, we assayed LegC3ΔTM’s ability to cause the release of a soluble lumenal protein during *in vitro* fusion. An assay to measure the extent of lysis during fusion has been previously developed, employing a strain of *S. cerevisiae* expressing a soluble green fluorescent protein (GFP) targeted to the vacuole lumen. This assay was used to show that normal *in vitro* vacuole fusion is non-leaky, but disruptions in the precise balance of SNARE proteins during fusion can lead to both fusion inhibition and vacuolar lysis [50]. With this assay, we can detect a background release of approximately 23% of the total available GFP over the course of a standard vacuole fusion reaction (Fig. 2D, bar 2); this release is fusion independent, in accordance with the previous study (bar 2 *vs* 4). As a positive control for vacuolar lysis induced by the fusion pathway, we added the soluble vacuolar SNARE, Vam7p, and confirmed the previously-reported release of GFP in the reaction supernatant (bar 6, [50]); this Vam7p-mediated lysis is completely fusion-dependent, as expected (bar 8). In the presence of inhibitory concentrations of LegC3ΔTM protein, we observe no additional GFP release over the standard background lysis (bars 2 *vs* 10). Therefore, LegC3ΔTM does not appear to inhibit the *in vitro* vacuole fusion reaction by causing lysis of the vacuoles.

The vacuole fusion assay used in this work depends upon the proper activation and activity of the Pho8p alkaline phosphatase. Therefore, it was essential to determine whether LegC3ΔTM inhibited the assay readout components, rather than the fusion pathway itself. For this test, we performed standard fusion reactions, but first solubilized vacuoles in Triton X-100. This allows for the activation of Pho8p via proteolytic cleavage in solution, independent of membrane fusion. Under these conditions, α-Vam3p or 3.5 µM LegC3ΔTM protein did not alter the activation of Pho8p in solution (Fig. 2E, bars 1 *vs* 5 *vs* 7). As expected, this solution activation of Pho8p was completely sensitive to the presence of the proteinase B inhibitor, Pbi2p (white bars). Taken together with the lysis data, these data show that LegC3ΔTM does not appear to disrupt the fusion assay through ‘off-pathway’ mechanisms, and likely has a biochemical effect on the regulation of vacuolar membrane fusion.

### LegC3ΔTM Associates with Vacuoles in the Absence of Transmembrane Domains

**Figure 3:**
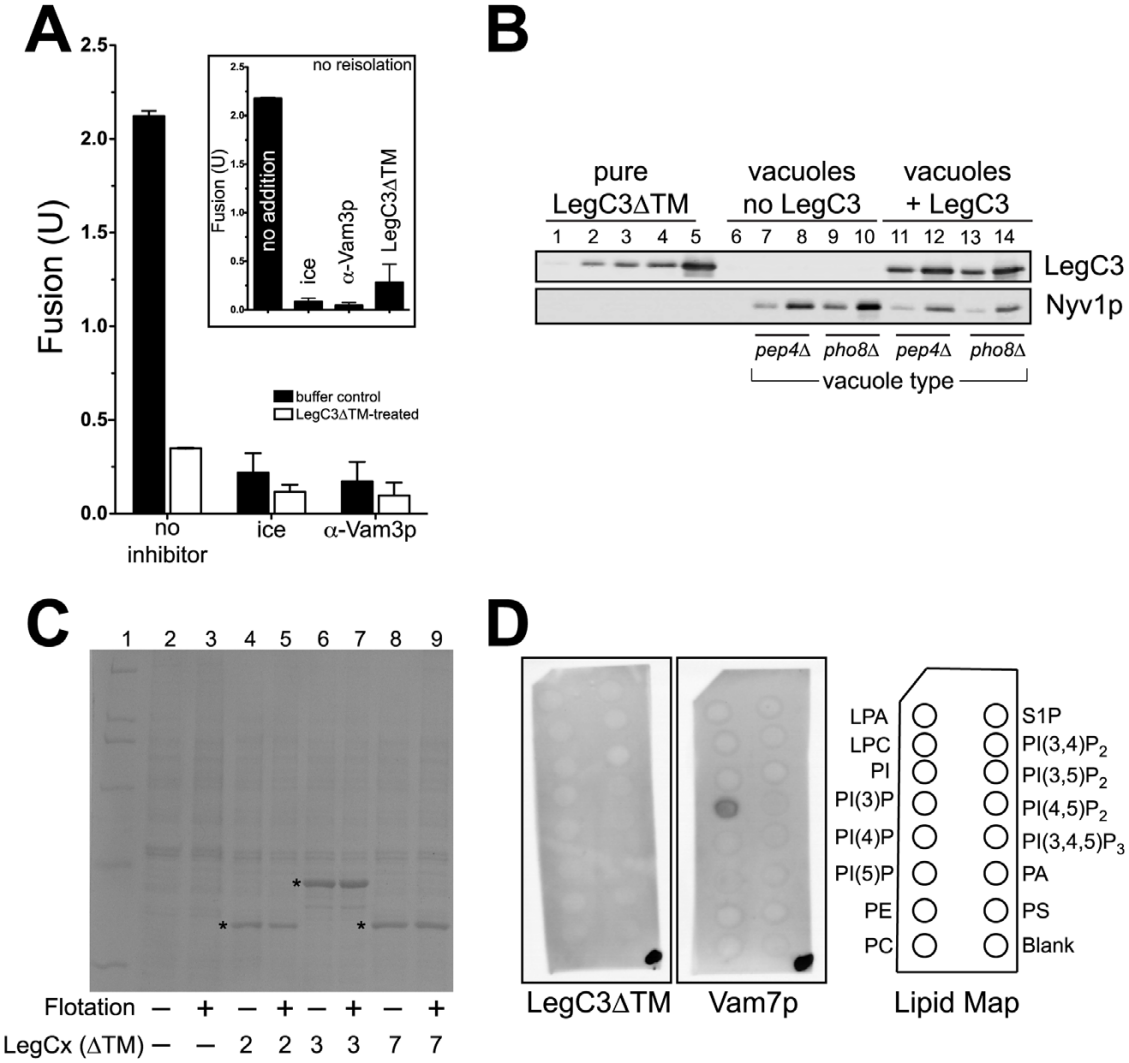
LegC3 associates with the vacuole in the absence of a transmembrane domain, inhibiting membrane fusion. (***A***) 300 µl reactions containing 200 µg vacuoles purified from either BJ3505 or DKY6281 were incubated at 27°C for 15 min, in either the absence or presence of 3.5 µM LegC3ΔTM protein. All incubations were performed in PS buffer (20 mM Pipes-KOH pH 6.8, 200 mM sorbitol). After incubation, vacuole mixtures were mixed with a 15% (w/v) Ficoll solution in PS buffer to obtain a final concentration of 8% (w/v) Ficoll, then added to a 11×34 mm Thickwall polyallomer tube (Beckman). Discontinuous steps consisting of 8%, 4%, and 0% Ficoll (PS buffer) layers were overlayed to fill the remaining volume in each tube. Vacuoles were reisolated via flotation in a TLS-55 swinging bucket rotor (Beckman, 134,000×*g*, 30 min, 4°C). Standard fusion reactions were performed from matching pairs of vacuoles that either had not been incubated (black bars), or had been incubated (white bars) with LegC3ΔTM. Due to day-to-day variability, fusion for each reisolated pair was normalized to the mean of the “no inhibitor” reactions (no LegC3∶2.11±1.06 U, *n* = 4;+LegC3∶0.35±0.11 U, *n* = 4); error is standard deviation from mean. Standard low-salt fusion reactions were performed from these same vacuoles without reisolation, to show LegC3ΔTM inhibition (inset). These reactions were normalized to the mean of the “no addition” reactions (2.18±0.71 U, *n = *3); error is standard deviation from mean. (***B***) 0.25 µg and 1.0 µg of each refloated vacuole type were separated on SDS-PAGE, along with a standard curve (10, 50, 100, 200, 500 ng) of purified LegC3ΔTM protein. LegC3ΔTM and the vacuole-bound SNARE Nyv1p were detected with standard immunoblotting techniques. Estimation of LegC3ΔTM protein on vacuoles was carried out via software-based densitometry (ImageJ v. 1.43u, http://rsb.info.nih.gov/ij). (**C**) 100 µg yeast vacuoles isolated from BJ3505 were incubated (15 min, 27°C) without, or with 3.5 µM purified LegC2ΔTM, LegC3ΔTM, or LegC7ΔTM, in PS buffer, to a final volume of 300 µl. Samples were refloated as in (**A**), and equal fractions of vacuolar material prior to, and after, reisolation were separated via SDS-PAGE, and visualized with Coomassie staining. Purified Leg proteins are denoted with an asterisk. (**D**) Individual PIP Strip™ membranes (Echelon Bioscience) were used via suggested Manufacturer’s protocol, and incubated with 0.5 µg/ml purified LegC3ΔTM (right) or Vam7p (left). After washing, proteins were detected via standard immunoblotting techniques. Prior to blocking the membrane, 0.5 µl of the appropriate protein solution was applied to the lower-right corner of the membrane, to confirm antibody activity. Lipid abbreviations: LPA, lysophosphatidic acid; LPC, lysophosphocholine; PI, phosphatidylinositol; PI(3)P, PI 3-phosphate; PI(4)P, PI 4-phosphate; PI(5)P, PI 5-phosphate; PE, phosphatidylethanolamine; PC, phosphatidylcholine; S1P, sphingosine-1-phosphate; PI(3,4)P_2_, PI 3,4-bisphosphate; PI(3,5)P_2_, PI 3,5-bisphosphate; PI(4,5)P_2_, PI 4,5 bisphosphate; PI(3,4,5)P_3_, PI 3,4,5-trisphosphate; PA, phosphatidic acid; PS, phosphatidylserine.

To test the ability of LegC3ΔTM to associate with the yeast vacuole, both vacuole types were briefly incubated with inhibitory concentrations of LegC3ΔTM (3.5 µM, Fig. 3A, inset), and were subsequently reisolated via flotation. LegC3ΔTM was seen to strongly associate with the vacuole membrane (Fig. 3B). The vacuolar homotypic fusion R-SNARE, Nyv1p, was detected as a control for vacuolar loading of each vacuole type (*pep4*Δ *vs pho8*Δ). Based on software densitometry, approximately 200 ng LegC3ΔTM protein associated with 0.25 µg BJ3505 vacuoles, and 150 ng with 0.25 µg DKY6281, providing an estimate of the final LegC3ΔTM concentration at 3.3 µM in a standard vacuole fusion reaction, if made from these reisolated vacuoles (3 µg each vacuole type). Vacuoles reisolated from LegC3ΔTM-containing conditions show approximately 6-fold less fusion than vacuoles not incubated with LegC3 (Fig. 3A), and are inhibited nearly to levels comparable with routine inhibitors of the vacuole fusion pathway.

To show that the significant association of the LegC3ΔTM protein with the yeast vacuole does not inhibit fusion through a non-specific activity, the non-inhibitory LegC2ΔTM and LegC7ΔTM effector proteins were incubated with yeast vacuoles, and reisolated via flotation. As seen in Figure 3C, LegC2ΔTM, LegC3ΔTM, and LegC7ΔTM are found to associate with the vacuolar membrane to similar amounts after reisolation (asterisks), yet equimolar concentrations of LegC2ΔTM and LegC7ΔTM fail to inhibit fusion (Fig. 2B,C). Additionally, we observed that LegC3ΔTM protein does not appear to directly bind purified lipids, as assayed by incubations with a PIP Strip™ (Echelon Bioscience) containing 15 different, physiologically-relevant lipid species (Fig. 3D). As an assay control, the soluble SNARE Vam7p was found to bind solely to phosphatidylinositol 3-phosphate via its PI(3)P-binding PX domain (Fig. 3D, [65]). Therefore, the LegC3ΔTM interaction with the vacuole likely inhibits fusion, and while this activity does not appear to be a non-specific, mass lipid-binding effect, further characterization of LegC3ΔTM’s association with mixtures of purified lipids in authentic bilayers and liposomes is needed to completely rule out LegC3ΔTM lipid-binding activity.

### LegC3-mediated Fusion Inhibition is Reversible

In an effort to begin to determine the mechanism by which the *L. pneumophila* LegC3 protein may be inhibiting a eukaryotic SNARE-, Rab GTPase-dependent membrane fusion pathway, we took advantage of the fact that we can simply add many purified reagents directly to our vacuole fusion assays in the presence of the inhibitor, and measure fusion responses. In many cases, inhibitors of the vacuole fusion assay can be “rescued” by the addition of several different purified reagents, such as Sec17p [49], the soluble SNARE Vam7p [66], and yeast cytosolic extracts [67], providing clues to the inhibitor’s activity in the membrane fusion assay. We therefore attempted to rescue LegC3-mediated vacuole fusion inhibition with a variety of important soluble reagents.

**Figure 4:**
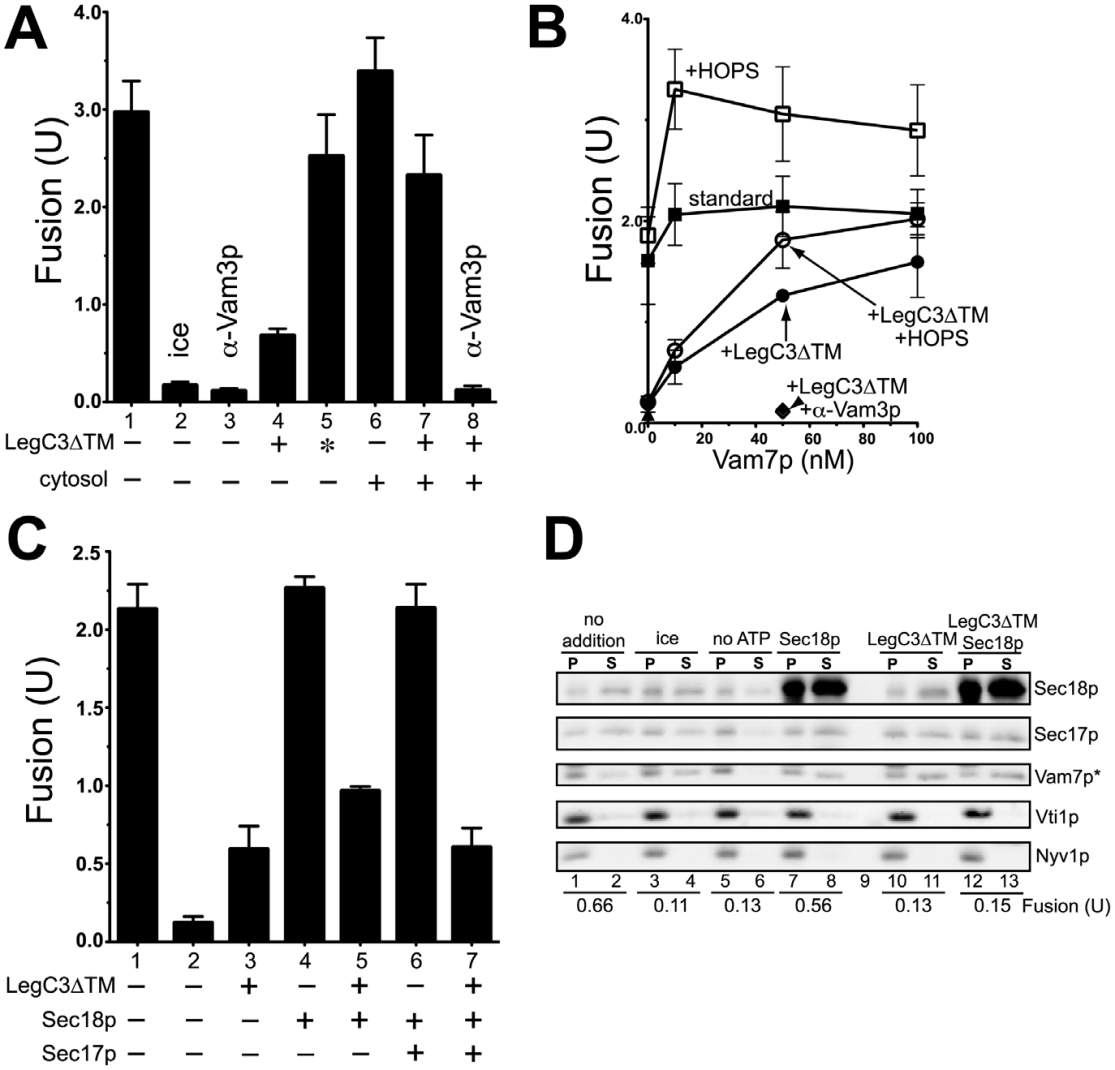
Yeast cytosol and a soluble vacuolar SNARE bypass LegC3-mediated fusion inhibition, but not SNARE chaperones. (***A***) Low-salt vacuole fusion reactions were incubated with either LegC3ΔTM protein (3.5 µM), heat-inactivated LegC3ΔTM (asterisk, 98°C, 15 min), yeast cytosol (31 µg), or α-Vam3p. All reactions lacking LegC3ΔTM contained no-protein buffer. Yeast cytosol was prepared from a phosphatase-deficient yeast strain, as in [93]. (***B***) Low-salt fusion reactions containing either 3.5 µM LegC3ΔTM (circles) or no-protein buffer (squared) were incubated with increasing concentrations of purified Vam7p SNARE protein; with (open symbols) or without (closed symbols) 20 nM purified HOPS complex. (***C***) Low-salt fusion reactions contained either LegC3ΔTM (3.5 µM), Sec17p (80 nM), or Sec18p (80 nM). Reactions lacking LegC3ΔTM contained the appropriate buffer control. All fusion reactions in (***A–C***) were performed in triplicate; error is standard deviation. (***D***) 3×volume (90 µl) low-salt fusion reactions were incubated at either 0°C or 27°C for 30 min with LegC3ΔTM buffer control, 80 nM Sec18p, 3.5 µM LegC3ΔTM, or without ATP. After incubation, reactions were placed on ice for 5 min. One 30-µl aliquot from each reaction was used to assay fusion standard procedures, and one 30 µl aliquot from each reaction was diluted with 30 µl ice-cold PS buffer, and centrifuged to pellet membranes (6200×*g*, 6 min, 4°C). Supernatants were harvested, and equal portions of each supernatant (S) and vacuole pellet (P) were separated via SDS-PAGE. Proteins were detected by immunoblotting; Vam7p detection required a longer exposure time than the other listed proteins (asterisk). Blot and fusion values corresponding to this blot are representative of three independent experiments.

As seen previously, 3.5 µM LegC3 strongly inhibits vacuole fusion (Fig. 4A, compare bars 4 and 1); this inhibitory activity is abrogated through heat-inactivation of LegC3ΔTM (bar 5 *vs* 4). While the inclusion of total soluble yeast proteins provides only a modest stimulation to standard fusion (bar 6 *vs* 1), this addition can almost completely reverse the fusion inhibition induced by LegC3ΔTM protein (bar 7 *vs* 4). Importantly, this cytosol-based reversal of LegC3ΔTM requires the activity of vacuolar SNARE proteins, as antibodies against the critical syntaxin, Vam3p, completely block the ability of yeast cytosol to reverse LegC3ΔTM (bar 8 *vs* 7), suggesting the cytosolic reversal activity must proceed through the authentic, SNARE-dependent membrane fusion pathway.

The addition of Vam7p to purified vacuoles leads to rapid vacuolar docking [66], enhanced trans*-*SNARE complex formation [68], and subsequent vacuolar fusion. This activity enables exogenous Vam7p additions to bypass some inhibitors of the fusion pathway that would otherwise prevent the formation of critical trans-SNARE complexes across apposed vacuoles, such as α-Sec17p and the lack of ATP [66]. Concentrations of Vam7p up to 100 nM have little effect on the normal fusion reaction (Fig. 4B, closed squares), yet can almost fully reverse the LegC3ΔTM fusion block (closed circles). The yeast HOPS complex has affinities for SNARE complexes, phosphoinositides, and the Vam7p PX domain [69], and by virtue of these activities, enhances the activity of Vam7p’s ability to catalyze additional trans-SNARE complexes [68]. While HOPS cannot reverse LegC3-mediated fusion inhibition alone, it does provide enhancement of the Vam7p-mediated fusion reversal of LegC3 (open circles), suggesting that Vam7p trans-SNARE complex-forming activity is essential for the bypass of LegC3 inhibition. As was seen for the cytosol reversal, this Vam7p activity depends on SNARE complex formation, as judged by the sensitivity to α-Vam3p (diamonds).

Because Vam7p is known to drive vacuole fusion under conditions that prevent the required remodeling of initial cis to trans-SNARE complexes (termed “priming”) by the SNARE chaperones, Sec17p and Sec18p, we assayed whether LegC3 was inhibiting this initial SNARE priming step. Providing excess Sec18p, Sec17p, or Sec18p and Sec17p together, however, were unable to bypass LegC3-mediated fusion inhibition (Fig. 4C). In addition, LegC3 did not inhibit the ATP-dependent release of Vam7p and Sec17p from the vacuole membrane (Fig. 4D), a known hallmark of SNARE-chaperone dependent priming [16]; these concentrations of LegC3 continued to block vacuole fusion. Taken together, these data provide evidence that LegC3 does not inhibit the initial remodeling of SNARE complexes during priming stages, but may be inhibiting fusion by blocking the formation of trans-SNARE complexes across apposed vacuolar membranes, as evidenced by the ability of Vam7p to rescue fusion in the presence of LegC3.

### LegC3 Blocks Trans-SNARE Complex Formation during Vacuole Fusion

To measure the formation of trans-SNARE complex formation during vacuole fusion, we assay the physical interaction of an epitope-tagged SNARE on one vacuole with its partnering SNARE on the apposing vacuole. This modified fusion assay uses a vacuole harboring the Vam3p SNARE tagged with an internal calmodulin-binding peptide (CBP), but lacking the fusion-critical R-SNARE, Nyv1p. When these vacuoles are fused to vacuoles containing the full complement of SNARE proteins, CBP-Vam3p:Nyv1p interactions can be detected via calmodulin-affinity pull-downs and subsequent immunoblotting. Because CBP-Vam3p cannot interact with Nyv1p in cis prior to a fusion event, the isolation of Nyv1p with CBP-Vam3p in this assay scheme is indicative of successful trans-SNARE pairing prior to lipid mixing. Importantly, these modified vacuoles fuse with normal fusion kinetics and remain sensitive to our routine fusion inhibitors, suggesting that this assay remains a good model for physiological, SNARE-dependent membrane fusion [68].

**Figure 5:**
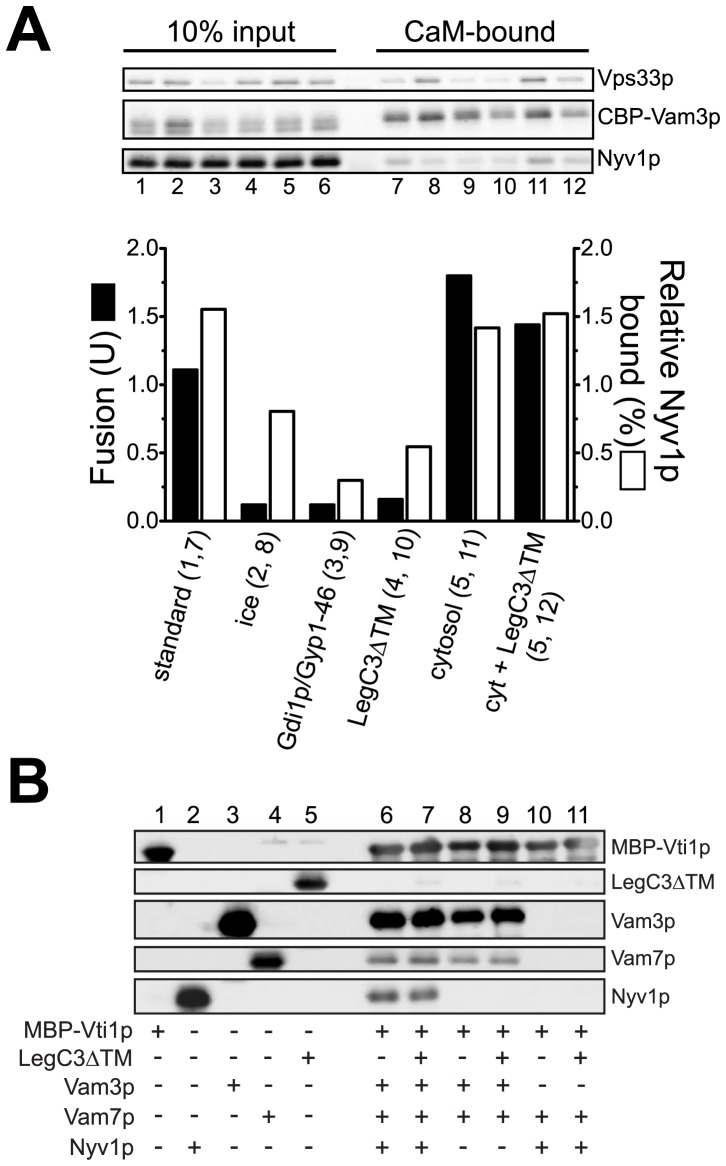
LegC3 blocks trans*-*SNARE complex assembly during fusion, but not directly. Vacuoles from BJ3505 *nyv1*Δ *CBP-VAM3* and DKY6281 were used to detect trans*-*SNARE complex formation during fusion, as described in [68]. Interactions between CBP-Vam3p Q-SNARE and Nyv1p R-SNARE are indicative of a proper trans-SNARE complex assembly. (***A***) Reactions contained either appropriate LegC3ΔTM buffer control, 1 µM Gdi1p and 1 µM Gyp1-46, 3.5 µM LegC3ΔTM, or 31 µg yeast cytosol; fusion (black bars) and trans*-*SNARE complex status (white bars) were assayed after 45 min. Vam3p and Nyv1p detections were carried out via standard immunoblotting techniques. Percent Vam3p and Nyv1p recovered with calmodulin affinity beads (Stratagene) were quantified via software-based densitometry (ImageJ), and by comparing to respective 10% input lanes. In the standard fusion condition, 17.1% of the total CBP-Vam3p was recovered, and this value was set to 1. Relative Nyv1p bound to CBP-Vam3p was determined by the following: Relative Nyv1p bound = (17.1%/Percent Vam3p recovered)×(percent Nyv1p recovered). Blot and fusion values are representative of two independent experiments. (***B***) Purified cytosolic domains of the four vacuolar fusion SNARE proteins, MBP-Vti1p, Vam3p, Vam7p, and Nyv1p (Experimental Procedures) were mixed together at a final concentration of 1 µM each in SSB buffer (20 mM HEPES, pH 7.4, 300 mM NaCl, 10% glycerol (v/v), and 0.1% Triton X-100 (v/v)). Equimolar concentrations of LegC3ΔTM protein were added, where indicated. The protein mixtures were allowed to assemble SNARE complexes on ice, without mixing, for 16 h. MBP-Vti1p was precipitated via standard amylose bead incubations (50 µl of 50% slurry in SSB), beads were washed 4 times with SSB, and eluted with 60 µl SSB +50 mM maltose. Eluates were separated via SDS-PAGE, and proteins were detected via immunoblotting.

When assayed for fusion and CBP-Vam3p:Nyv1p interactions after 45 min, vacuoles under standard fusion conditions were estimated to contain approximately 1.5% of the total Nyv1p in trans associations with the precipitated CBP-Vam3p protein (Fig. 5A, top, lanes 1 and 7, graphed below). About half that amount of trans-SNARE pairs was formed when vacuoles were left on ice, yet fusion was dramatically reduced (top, lanes 2 and 8, graph below). This result is not surprising, as the temperature-sensitive step of fusion occurs downstream of trans-SNARE pairing [70]. Extracting the vital Rab-family small GTPase, Ypt7p, from the vacuolar membrane with the Rab protein GDP-dissociation inhibitor, Gdi1p, and the Rab GAP protein, Gyp1-46p, [53] inhibits the formation of trans-SNARE interactions, with correspondingly low levels of fusion (top, lanes 3 and 9, graph below). In the presence of LegC3ΔTM protein, trans-SNARE pairs are formed at levels only slightly higher than Gdi1p/Gyp1-46 treatment, and fusion remains strongly inhibited (top, lanes 4 and 10, graph below). When present in levels that can bypass LegC3-mediated inhibition, yeast cytosol does not alter the total amount of trans-SNARE pairs formed during fusion (top, lanes 5 and 11), yet increases the total amount of trans-SNARE pairs formed in the presence of LegC3 to normal levels (top, lanes 6 and 12). This cytosol-dependent restoration of trans-SNARE complexes in the presence of LegC3 results in restored vacuole fusion (Fig. 4A, bottom). Thus, LegC3 protein inhibits the formation of trans-SNARE pairs during homotypic vacuole fusion, thereby preventing vacuolar lipid and content mixing.

A previous report suggested that LegC3 may have SNARE-like motifs [62], therefore interacting with eukaryotic SNAREs and inhibiting specific eukaryotic membrane fusion events, as was determined for the IncA protein from *Chlamydia trachomatis* [62], [64]. To test whether LegC3 was directly interacting with the known vacuolar SNAREs directly involved with homotypic vacuole fusion, and thereby preventing SNARE complex formation, we assayed the ability of the soluble SNARE domains of these proteins to assemble into physiologically-relevant SNARE complexes in solution, either in the presence or absence of the LegC3ΔTM protein.

SNARE complexes are formed through the assembly of four “SNARE motifs” into the characteristic parallel, coiled-coil structure [71]. Genetic and structural studies of SNARE interactions have noted that functional SNARE complexes assemble with a central ionic core termed the 0-layer [72], [73], comprised of 3 glutaminyl residues and one arginyl residue, each donated from a single SNARE domain. The residue at this location in the SNARE protein denotes whether a SNARE is classified as a “Q-SNARE” or an “R-SNARE.” Normal homotypic vacuole fusion requires that yeast vacuolar SNAREs follow this assembly format, termed the “3Q:1R rule” [74]. The SNARE domains of the three vacuolar Q-SNAREs (Vam3p, Vti1p, Vam7p; Q_a_-, Q_b_-, and Q_c_-SNARES, respectively) and one R-SNARE (Nyv1p) readily form SNARE complexes in solution, as determined by co-precipitation with epitope-tagged MBP-Vti1p (Fig. 5B, lane 6). The addition of equimolar LegC3ΔTM to this mixture does not affect the assembly of this vacuolar SNARE complex in solution (Lane 7). To test whether LegC3ΔTM could take the place of either an R-SNARE or a Q-SNARE in this vacuolar complex, we omitted one of each type of vacuolar SNARE. While a stable “3Q” vacuolar SNARE complex is known to form in solution ([52], Fig. 5B, lane 8), LegC3ΔTM does not prevent this assembly, nor associate with this complex (lane 9). SNARE complexes consisting of a 2Q:1R 0-layer do not form (lane 10); LegC3ΔTM does not play the role of a Q_a_-SNARE in this structure by replacing Vam3p and enhance assembly (Lane 11), nor does it associate with any of the soluble SNARE complexes. Additionally, LegC3ΔTM did not associate with any of these four vacuolar fusion SNAREs directly in solution (data not shown). Thus, it is unlikely that LegC3 is directly interacting with these particular vacuolar SNAREs as a bacterial “inhibitory” SNARE-like protein, although we cannot test whether the SNARE complexes formed in solution can associate with regulatory proteins like those embedded in lipids, or whether these solution-formed SNARE complexes are in the proper conformation for productive fusion.

### LegC3 does not Inhibit the Core Vacuolar Membrane Fusion Machinery

The core SNARE-dependent membrane fusion biochemistry has been successfully reconstituted via a synthetic lipid-mixing assay, utilizing the lipid-associated, FRET-based quenching fluor pair, NBD-PE (*N*-(7-nitro-2,1,3-benzoxadiazole-4-yl)-phosphatidylethanolamine) and Rh-PE (*N*-(lissamine rhodamine B sulfonyl) phosphatidylethanolamine) [75]. When these probes are incorporated together into the same bilayer membrane at sufficient concentration, the emission of the NBD-PE probe is quenched by the nearby Rh-PE, providing low fluorescent signals upon excitation of the NBD moiety. Upon successful fusion of these labeled liposomes to unlabeled liposomes, fluorescence quenching decreases, and an increase in NBD fluorescence can be assayed. This fluorescence increase is therefore a direct measure of lipid mixing and fusion.

Using this assay, synthetic proteoliposomes with a lipid composition mirroring that of the yeast vacuole and harboring the four full-length, transmembrane domain-containing vacuolar SNARE proteins involved in homotypic fusion, have been shown to rapidly mix lipid bilayers, but only in the presence of the SNARE chaperones Sec17/18p, regulatory lipids such as PI(3)P, and the HOPS complex, thereby recapitulating much of the known biochemical regulation of homotypic vacuole fusion [58], [76]. It is also known that proper SNARE complex remodeling and trans-SNARE complex formation must precede fusion in this assay [77], and as shown by others for the reconstitution of alternative SNARE-dependent lipid-mixing schemes [19], [78], [79]. Indeed, some bacterial proteins from intracellular pathogens have already been shown to inhibit the rate of membrane fusion in these synthetic SNARE-dependent lipid-mixing assays, thus highlighting the ability of some bacteria to directly modulate the core membrane fusion machinery *in vitro* [62]. We therefore employed this highly purified assay of membrane fusion to assay the potential function of *L. pneumophila* LegC3 on the regulation of core yeast vacuole membrane fusion machinery.

**Figure 6:**
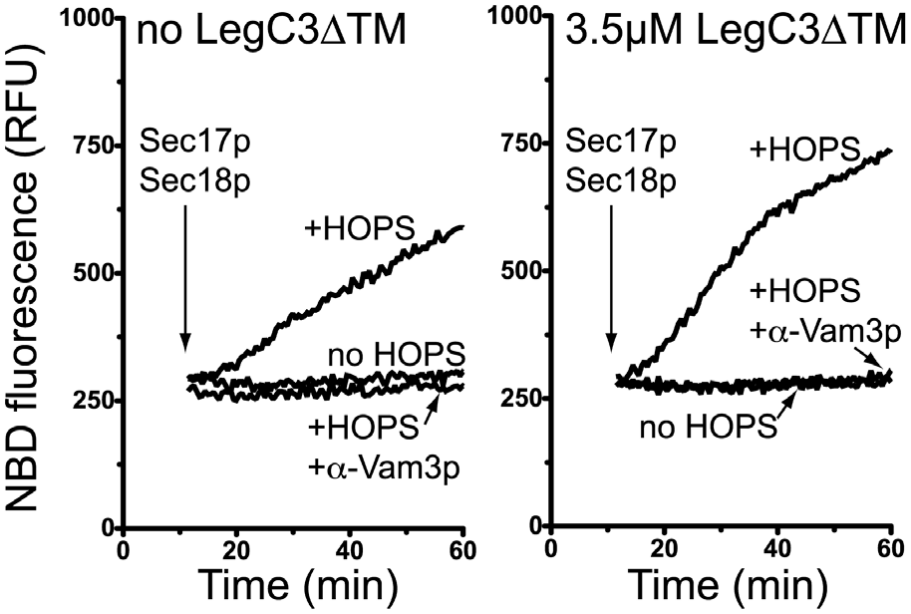
LegC3 does not inhibit purified SNARE-dependent lipid mixing. Proteoliposome lipid mixing assays were carried out as in Materials and Methods. Reactions containing 4-SNARE donor and acceptor proteoliposomes were performed in the absence (left) or presence (right) of 3.5 µM LegC3ΔTM. All reactions contained Sec17p and Sec18p; additional reaction constituents are indicated. Raw NBD fluorescence units were plotted as a measure of lipid mixing; graph is representative of three independent experiments.

‘Vacuole-mimic’ proteoliposomes bearing full-length Nyv1p, Vam3p, Vam7p, and Vti1p are unable to undergo lipid mixing in the absence of the HOPS complex (Fig. 6, left panel), in accord with several earlier works [58], [77]; Sec17p/18p-dependent disassembly of cis-SNARE complexes is insufficient to drive lipid-mixing under these conditions. Upon the addition of HOPS complex, however, these liposomes rapidly tether [80] and mix their lipid bilayers. Underscoring the requirement for functional trans-SNARE complexes to be formed prior to lipid-mixing, antibodies directed against the Vam3p SNARE abolish HOPS-dependent lipid-mixing. The addition of 3.5 µM LegC3ΔTM protein to this assay does not inhibit HOPS-dependent proteoliposome fusion, but rather has a modest – but reproducible – stimulatory effect that was not further studied (Fig. 6, right panel). Importantly, LegC3 does not induce proteoliposome fusion in the absence of the HOPS complex, or in the presence of α-Vam3p (right panel), showing that authentic SNARE-, SNARE chaperone-, and HOPS-dependent membrane fusion occurs in the presence of *L. pneumophila* LegC3 protein, and thus, LegC3 does not appear to inhibit SNARE-dependent membrane fusion by directly inhibiting the activity of the core vacuolar SNARE-dependent membrane fusion machinery.

## Discussion

Upon invasion of host eukaryotic cells, pathogenic intracellular bacteria must evade a number of host cell defenses for continued survival. One such evasion mechanism entails preventing the fusion of the bacteria-laden phagosome with the host degradative lysosome. The direct modulation of these membrane trafficking pathways has been noted for several important pathogens, including *Salmonella enterica* [81], *Chlamydia trachomatis* [82], and *Legionella pneumophila* [31]. A number of secreted effector proteins from these pathogens have been recently discovered to modulate a number of eukaryotic vesicular trafficking and membrane fusion pathways, some of which may be playing a direct role in the inhibition or the delay of host phagolysosomal fusion. Using a simple *in vivo* model system of a eukaryotic cell, the budding yeast *Saccharomyces cerevisiae*, de Felipe et al. identified three known secreted effector proteins from *L. pneumophila* that induced endomembrane trafficking, protein sorting, and intracellular membrane morphology defects upon ectopic expression in yeast [42]. One of these proteins, LegC3, trafficked to the yeast vacuole and plasma membranes, and induced extensive fragmentation of the intracellular degradative vacuole. In addition, LegC3 showed evidence of inhibiting *in vivo* endosome:lysosome fusion pathways when expressed in the soil-living amoeba, *Dictyostelium discoideum*, suggesting that LegC3 may be a component of *Legionella*’s ability to directly modulate host membrane trafficking pathways or fusion events during pathogenesis, thereby providing some protection from lysosomal degradation. The mechanisms by which LegC3 could potentially alter membrane fusion events *in vivo* were left unclear, although this protein’s structure may be “SNARE-like” [42], [62], thereby providing a possible link to a direct role in the modulation of eukaryotic membrane trafficking and fusion events.

In this work, we describe a functional biochemical assay for LegC3 activity. This protein was shown to inhibit a model of eukaryotic membrane fusion with similarities to mammalian lysosomes, the homotypic fusion of the yeast vacuole. Like many similar proposed intracellular membrane fusion pathways, including those of neurotransmission, exocytosis, and ER-Golgi trafficking pathways, vacuole fusion relies on the precise spatiotemporal regulation of a number of conserved lipids and proteins, including phosphoinositides, SNAREs, Rab-family GTPases, tethering factors, and SNARE protein chaperones [43]. Factors influencing one or more members of this core fusion machinery also may correspondingly regulate the process of fusion. We now provide evidence the LegC3 protein inhibits yeast vacuole fusion *in vivo*, as ectopic expression of *LEGC3* induces extensive vacuolar fragmentation, resulting in the growth sensitivity to exogenous zinc and caffeine (Figure 1). These phenotypes are indicative of vacuoles which are defective for homotypic fusion, although this is not necessarily caused through a direct inhibition of membrane fusion. An extensive screen of the non-essential yeast knockout collection [44] identified a number of mutations that lead to fragmentation of the yeast vacuole, and many of them, like the Arf GAP Glo3p, the clathrin light chain subunit Clc1p, and Vps3p, a subunit of the endosomal CORVET tethering complex, are unlikely to be directly involved in vacuole:vacuole fusion, and more likely involved in trafficking the proper fusion machinery components to the vacuole. While it is clear that LegC3 trafficks to the vacuole upon expression in *S. cerevisae* ([42], and data not shown), the protein signals that direct LegC3 to the yeast endomembrane system upon expression remain unclear. A functional N-terminal eukaryotic ER import signal could not be detected in the primary sequence of LegC3 (SignalP, v3.0, www.cbs.dtu.dk/services/SignalP/), although up to 20% of random 20-mers could be active as ER import signals [83]. Additionally, no other known consensus eukaryotic subcellular localization signals could be detected (TargetP, v.1.1, www.cbs.dtu.dk/services/TargetP). During the pathogenesis of *L. pneumophila*, however, these signals are probably not necessary, as LegC3 will be likely secreted at the site of action and may simply function locally. The subcellular localization of LegC3 within the host cell during an active *L. pneumophila* infection, to the best of our knowledge, is not currently known.

Despite our best efforts, we were unable to obtain sufficient vacuolar material for fusion analysis from our *pep4*Δ vacuole fusion tester strains when expressing LegC3 *in vivo*. This was likely due to the extensive vacuolar fragmentation induced by LegC3 induction (Fig. 1). In order to better study the effects of LegC3 on membrane dynamics in yeast, therefore, we bypassed the trafficking requirements of *in vivo* studies by simply providing the purified LegC3 protein to *in vitro* assays of yeast homotypic vacuole fusion. In sufficient concentration, LegC3 shows a striking ability to inhibit the homotypic fusion of vacuoles (Fig. 2A), in support of the aforementioned *in vivo* phenotype. Importantly, this shows that the potential fusion inhibitory activity of LegC3 *in vivo* does not stem from the possibility of LegC3 blocking or altering normal fusion component traffic to the yeast vacuole *in vivo*, but rather results from a direct effect on some aspect of the fusion machinery. Additionally, the ability of LegC3 to inhibit fusion of the yeast vacuole *in vitro* does not appear to be shared by other coiled-coil proteins of *L. pneumophila*, LegC2/YlfB and LegC7/YlfA. Because LegC3 and LegC7 proteins were found to induce vacuolar protein mis-sorting and endomembrane morphology defects upon expression in yeast [42], it is likely that LegC3, LegC7, and possibly LegC2, share similar biological activities within the eukaryote. Phenotypic and subcellular localization differences during *in vitro* expression, however, suggest that the LegC2, LegC3, and LegC7 proteins likely have affinities for different protein receptors within eukaryotic membranes.

To support the possibility that LegC2, LegC3, and LegC7 proteins are involved in modulating different trafficking pathways, it is known that membrane fusion and trafficking machineries assigned to regulate individual fusion events are specific and compartmentalized; there is little sharing of SNAREs, Rab GTPases, and SM proteins throughout cellular trafficking pathways [84]. For example, SM proteins only complex with a specific set of cognate SNARE proteins during fusion – even when in the presence of noncognate SNAREs – and can regulate the assembly of only the proper SNARE complex, thereby driving fusion. Therefore, SM family member proteins are distinctly compartmentalized in the cell, and individual SM proteins cannot compensate for the absence of a different SM protein at another organelle [85]. Given the exquisite protein specificity of the fusion machinery, it seems likely that bacterial proteins that alter eukaryotic membrane trafficking should also do so with compartmental or organellar specificity.

Although it had been previously shown that LegC3 requires at least one (of two predicted) functional transmembrane domain to elicit its vacuolar trafficking and fragmentation defects *in vivo* [42], our experiments show that the *in vitro* membrane fusion inhibition caused by LegC3 does not explicitly require its insertion into a membrane via a transmembrane domain (Fig. 3). Therefore, LegC3’s predicted transmembrane domains are likely used to either properly traffic within host compartments, or to simply concentrate the protein at its site of action on internal eukaryotic membranes. The mechanisms by which intracellular pathogens can secrete transmembrane domain-containing proteins into their hosts are not completely understood, although *Legionella* appears to utilize typically-eukaryotic C-terminal, Ras GTPase-targeting C*AAX* prenylation motifs [86] and phosphoinositide-binding PH domains [87] for directing some secreted effectors to the surface of eukaryotic membranes. Previous studies on the translocation of LegC7/YlfA showed that the predicted hydrophobic transmembrane domain does not interfere with bacterial translocation, and does not immediately cause insertion into the LCV, as shown by time course microscopy [27]. In fact, LegC7/YlfA showed association with early secretory compartments that later became a part of the ER-derived replicative vacuole, suggesting that LegC7/YlfA – and possibly LegC2/YlfB – will be intimately involved in altering normal eukaryotic membrane traffic. Elucidation of the mechanisms by which pathogens secrete and insert integral transmembrane proteins into host membranes may provide new insights into a potentially critical component of bacterial pathogenesis.

We have also shown that the inhibitory effects of LegC3 on a eukaryotic SNARE- and Rab-family GTPase-dependent membrane fusion reaction is reversible by a soluble vacuolar SNARE protein, Vam7p, which is known to induce increased levels of trans-SNARE complexes during the fusion pathway (Fig. 4). Indeed, LegC3 appears to inhibit the formation of the proper trans-SNARE complexes during homotypic vacuole fusion (Fig. 5), providing direct evidence that LegC3 may be interfering with the normal pathways of organellar membrane fusion. At present, we do not know the target or receptor of LegC3 on the yeast vacuole for fusion inhibition, but it is unlikely to be either the four essential homotypic vacuole fusion SNAREs (Fig. 5B, Fig. 6), or the Sec17p and Sec18p SNARE chaperones required for cis- and trans-SNARE complex remodeling prior to fusion (Fig. 4 C and D). If LegC3 is behaving as a bacterial SNARE mimic, as has been suggested [62], [88], the putative SNARE complexes containing LegC3 on the vacuolar membrane are not targets for disassembly by the essential Sec18p AAA+ ATPase, as Sec17p/Sec18p activity cannot reverse the fusion inhibition caused by LegC3 (Fig. 4C). While we did not detect direct association of LegC3 with purified yeast vacuolar 4-SNARE, 3Q-SNARE, or 2Q:1R SNARE complexes (Fig. 5B), it is possible that LegC3 enhances improper assembly of some of the homotypic vacuole fusion SNARE proteins into complexes with other SNAREs present on the vacuole, such as Ykt6p or Pep12p. These other SNAREs are known to physically interact in complexes with some of the vacuole fusion SNAREs, but are thought to be incapable of directing homotypic vacuole fusion [89], [90]. In addition, reversal of LegC3-mediated inhibition of vacuole fusion can be achieved through the introduction of soluble yeast cytosolic proteins. Although Vam7p would exist in this cytosolic mixture, previous calculations suggested Vam7p to exist at ∼100 nM within the cytosol [66], providing a final concentration of approximately 3.3 nM, when diluted into our reaction mixtures; this concentration of purified Vam7p fails to reverse LegC3ΔTM inhibition (Fig. 4). While this suggests the existence of a higher-affinity target of LegC3 in the yeast cytosol, we cannot rule out the possibility that the cytosolic reversal is mediated via the presence of Vam7p, and further studies are required to confirm this. Regardless, the presence of a reversal activity in yeast cytosol may also help explain the report that cytosolic LegC3 derivatives lacking transmembrane domains are unable to induce vacuolar morphology defects upon expression in *S. cerevisiae* [42].

We found that in a highly-purified biochemical model system for vacuolar membrane fusion, LegC3ΔTM was unable to inhibit the mixing of synthetic membranes catalyzed by the vacuolar homotypic fusion SNAREs, Sec17p, Sec18p, and the HOPS complex (Fig. 6), which was in striking contrast to the results obtained while using the *in vitro* vacuole fusion assay. This synthetic vesicular lipid-mixing assay is clearly inhibited by direct inhibitors of the vacuole fusion pathway such as α-Vam3p and the lack of Sec17p/18/HOPS complex activities [this study and [58]]. Our data show that these critical fusion proteins on the yeast vacuole are likely not the direct targets of LegC3 protein, and that other protein or lipid factors found on the yeast vacuole may be responsible for the LegC3-mediated fusion inhibition that we observe on vacuoles. It also remains possible that while the LegC3ΔTM protein was able to inhibit the fusion of vacuoles without direct membrane association, LegC3 may only inhibit this lipid-mixing assay when incorporated into the bilayer membrane, as was the case for the ability of IncA to inhibit mammalian SNARE-mediated lipid-mixing events [62]. Efforts are currently underway to identify the vacuolar and cytosolic targets of LegC3, as discovery of these targets will be critical for understanding the mechanisms by which *L. pneumophila* LegC3 inhibits yeast SNARE-dependent membrane fusion. As LegC3 has been shown to modulate membrane traffic in yeast, protozoan, and mammalian cells [42], analysis of LegC3 within the context of a facile and proven yeast model membrane fusion system should provide additional insights into the molecular mechanisms of LegC3 activity during *Legionella* pathogenesis. Taken together, LegC3 likely plays a role in at least misdirecting membrane traffic within the host by directly preventing specific membrane fusion events, such as the fusion of the bacteria-laden phagosome with endosomal or lysosomal compartments, and thus helping ensure intracellular survival of the pathogenic organism.
